# Factors modulating the impact of the COVID-19 pandemic on posttraumatic stress symptomatology of the Spanish healthcare workers: A cohort study

**DOI:** 10.1371/journal.pone.0323777

**Published:** 2025-06-16

**Authors:** Beatriz Arregui-Gallego, María Isabel Orts-Cortés, María Teresa Moreno-Casbas, Eva Abad-Corpa, Rafaela Camacho-Bejarano, Mª Ángeles Cidoncha-Moreno, Isabel Feria-Raposo, Javier Iruzubieta-Barragán, Montserrat Carmona, Estíbaliz Cristóbal-Domínguez, Leticia Bernués-Caudillo, Elvira Casado-Ramírez, Alda Recas-Martin, Dolores Sánchez-López, M. Consuelo Company-Sancho, Noelia López Rascón, Silvia Esteban-Sepúlveda, María Clara Vidal-Thomàs, Isabel Alonso, Daniel Muñoz-Jiménez, José Vicente Segura-Heras, Joaquín Moncho, Manuel Rich-Ruiz

**Affiliations:** 1 Research Unit of the Primary Care Assistance Management (GAAP) – Primary Care Biomedical Research and Innovation Foundation (FIIBAP), Madrid, Spain; 2 Nursing and Healthcare Research Unit (Investén-ISCIII), Instituto de Salud Carlos III, Madrid, Spain; 3 CIBER of Frailty and Healthy Ageing, (CIBERFES) Instituto de Salud Carlos III, Madrid, Spain; 4 Nursing Department, University of Alicante (BALMIS), Alicante Institute for Health and Biomedical Research (ISABIAL, Group 23), Alicante, Spain; 5 Institute Murciano for Biosanitary Research (IMIB). Nursing and Healthcare Research Unit ENFERAVANZA; Murcia Health Service. El Palmar, Murcia, Spain; 6 Department of Nursing, Nursing Faculty, University of Huelva, Campus El Carmen, Huelva, Spain; 7 Research Network on Chronicity, Primary Health Care and Health Promotion (RICAPPS), Madrid, Spain; 8 IIS Bioaraba, Prevention, Promotion and Health Care, Vitoria-Gasteiz, Spain; 9 Osakidetza, Directorate General of Osakideza, Assistant Directorate of Nursing, Vitoria-Gasteiz, Spain; 10 Academy of Nursing Sciences of Bizkaia, Bizkaia, Spain; 11 Benito Menni Mental Health Care Complex, Barcelona, Spain; 12 FIDMAG Germanes Hospitalaries Research Foundation, Barcelona, Spain; 13 CIBERSAM, Instituto de Salud Carlos III, Madrid, Spain; 14 Universidad Europea Miguel de Cervantes, Valladolid, Spain; 15 Health Technology Assessment Agency, Instituto de Salud Carlos III, Madrid, Spain; 16 IIS Bioaraba, Nursing and Healthcare Research Group, Vitoria-Gasteiz, Spain; 17 Osakidetza Servicio Vasco de Salud, OSI Araba, Vitoria-Gasteiz, Spain; 18 Ministerio de Sanidad, Madrid, Spain; 19 Gerencia Atención Primaria de Burgos, Burgos, Spain; 20 Health Promotion Service, Directorate General for Public Health, Canary Islands Health Service, Las Palmas de Gran Canaria, Spain; 21 Hospital General Universitario de Elche, Elche, Alicante, Spain; 22 Facultat d’Infermeria, Universitat de Barcelona, Barcelona, Spain; 23 Health Research Institute of the Balearic Islands (IdISBa), Balearic Islands Health Services, Servei de Salut de les Illes Balears, Balearic Islands, Spain; 24 Hospital Clínico San Carlos. IdISSC, Madrid Spain; 25 Instituto Universitario Centro de Investigación Operativa (CIO), Universidad Miguel Hernández, Elche, Spain; 26 Research Unit for the Analysis of Mortality and Health Statistics, Department of Community Nursing, Preventive Medicine, Public Health and History of Science, University of Alicante, Alicante Institute for Health and Biomedical Research (ISABIAL. Group 23), Alicante, Spain; 27 Maimonides Institute for Biomedical Research (IMIBIC), University of Cordoba (UCO), Reina Sofía University Hospital (HURS), Córdoba, Spain; Xiamen University - Malaysia Campus: Xiamen University - Malaysia, MALAYSIA

## Abstract

**Introduction:**

The COVID-19 pandemic generated a global health crisis that significantly impacted healthcare systems and professionals. Healthcare workers were exposed to high levels of psychological distress, including posttraumatic stress symptomatology (PTSS).

**Aim:**

Analyse the evolution of PTSS among Spanish healthcare workers during the COVID-19 pandemic, and to identify associated factors.

**Method:**

A multicenter prospective cohort study with a 12-month follow-up was conducted. PTSS was the primary outcome. Secondary variables included sociodemographic, occupational, psychological, and coping-related factors. Statistical analyses comprised bivariate comparisons and multivariate modelling, such as generalized linear models and linear mixed models.

**Results:**

Of the 428 participants, 180 completed the 12-month follow-up. At baseline, changes in work posts, negative family-work relations, avoidant coping, burnout symptoms, and emotional intelligence were associated with PTSS levels. Linear mixed models showed a significant decrease in PTSS over the 12-month period, regardless of gender, age, household type, occupational role, contract type, job title, level of care or type of service (p < 0.001). The generalised linear model explained 25.5% of the variance in PTSS levels at baseline, highlighting the role of psychological and coping factors over sociodemographic or occupational characteristics.

**Conclusions:**

This study highlights the need for early identification and intervention focused on psychological and coping mechanisms. Promoting emotional regulation, reducing burnout, and addressing maladaptive coping may help mitigate long-term psychological effects among healthcare workers during public health crises.

## Introduction

The COVID-19 pandemic has triggered an unprecedented global crisis, increasing the global burden of both physical and psychological disease [1]. Given the central role of healthcare workers (HCWs) in maintaining health system functionality, the increased work pressure they faced not only affected their health but also posed risks to the health of the general population [2,3]. This was supported by evidence linking impaired decision-making to maladaptive psychological responses [4]. Ensuring the physical and mental well-being of HCWs is thus essential to avoid system collapse and its negative consequences on population health [5].

Most of the current evidence on the psychological impact of the pandemic on HCWs stems from cross-sectional studies [6], and existing longitudinal studies report mixed findings regarding the persistence or evolution of mental health outcomes [7,8]. There was a pressing need for research that tracks the evolution of HCWs’ mental health over time and identifies factors that modulate psychological distress [2]. This knowledge was key to developing timely interventions and informing future public health emergency preparedness.

Since the onset of the COVID-19 pandemic in 2019, HCWs have faced rapidly changing public health measures [9], marked by uncertainty, lack of preparation, and high psychological demand [10]. They were often redeployed to overburdened units, forced to work under unfamiliar protocols and decision-making contexts, with limited access to resources and personal protective equipment (PPE) [11–13]. These challenges were compounded by fears of infection, ethical dilemmas, intense workloads, and social stigma from the public [14,15].

Public health restrictions introduced additional stressors, including social isolation, financial insecurity, and disrupted routines [16,17]. The loss of social support and lifestyle changes had been shown to negatively impact quality of life and increase psychiatric risk [10,12,14,18].

Post-traumatic stress disorder (PTSD) may emerge following highly stressful or traumatic events [19]. When symptoms did not meet diagnostic thresholds, they were referred to as post-traumatic stress symptomatology (PTSS), which often coexists with burnout [15,20]. HCWs, due to their prolonged exposure to trauma and stress during the pandemic, were a high-risk group for such psychological conditions [1,21,22].

Even before COVID-19, HCWs faced a mental health crisis marked by high rates of psychiatric symptoms and maladaptive coping strategies [23]. In addition to environmental factors, personal characteristics such as being younger, female, less experienced, or having a history of psychiatric disorders have been associated with increased vulnerability to PTSS, anxiety, and depression [24,25]. Gender disparities may also be heightened by the predominance of women in nursing roles, who provide direct patient care [18,26].

Psychological resilience – the capacity to adapt to adversity – was a protective factor against poor mental health outcomes [27]. Coping strategies also played a key role: problem-focused and approach-focused strategies are generally beneficial. In contrast, emotion-focused and avoidant strategies were associated with a higher risk of experiencing psychological distress [1,28]. Moreover, emotional intelligence, including self-awareness and emotional regulation, was closely linked to psychological well-being among HCWs [29,30].

The primary aim of this study is to analyze the evolution of PTSS among Spanish HCWs during the COVID-19 pandemic and to examine associated factors.

The secondary objectives are to identify healthcare workers at risk of developing symptoms related to PTSD; to study the evolution of PTSS throughout the COVID-19 pandemic; to provide information on how the working conditions influenced the Spanish healthcare workers’ post-traumatic stress disorder-related symptoms; to determine how the psychological, coping and socio-demographic characteristics of the HCWs influenced their PTSS.

## Methods

### Study design

#### Multicenter prospective cohort study with a 12-month follow-up.

##### Setting

The project was carried out in eight hospitals, five primary care centers, and four social and health care institutions. The participating institutions are distributed across the autonomous communities of Andalusia, the Balearic Islands, Castile-La Mancha, Castile and Leon, Catalonia, the Valencian Comunity, Madrid, the Basque Country, Murcia, and La Rioja. During the COVID-19 pandemic, these institutions faced considerable structural and organizational challenges. In hospital settings, bed capacity ranged from 400 to over 1,300 beds, with all centers expanding their ICU capabilities—some doubling or tripling their number of critical care beds. Several hospitals managed more than 800 COVID-19 admissions during the first wave alone. In primary care, although inpatient beds were not available, thousands of SARS-CoV-2 cases were monitored remotely, with some networks conducting over 1.5 million consultations monthly at peak times. Staff capacity was also expanded across settings through temporary hires, reassignments, and emergency reinforcements. For example, individual hospitals employed over 5,000 professionals, while primary care services in regions like Madrid and Murcia operated with more than 8,000 healthcare workers. Social and healthcare institutions adapted by creating internal COVID-19 units, managing hundreds of infected residents, and mobilizing large multidisciplinary teams. In each of the selected facilities, a researcher was responsible for recruiting and following up with the participants.

We performed recruitment through convenience sampling from February 3, 2021, to August 29, 2022. All study participants signed an informed consent form before the start of data collection.

### Participants

Inclusion criteria: HCWs aged 18 or older, including doctors (physicians), nurses, geriatric carers, and healthcare assistants (HCAs). Participants were required to have worked in any collaborating institution and to have provided care for SARS-CoV-2 infectious patients to patients with confirmed or suspected SARS-CoV-2 infections during the Spanish state of alarm (SA) which lasted from March 14 to June 21, 2020. Participants who met the inclusion criteria and had left their post for less than 30 days during the SA were also included.

Exclusion criteria: HCWs who screened positive for possible PTSD according to the Posttraumatic Stress Disorder Symptom Severity Scale-Revised (EGS-R) according to DSM-5 criteria [31] due to ethical reasons. These professionals were informed of the availability of specialized support.

### Variables and measurements

Data collection was carried out through online questionnaires using the Redcap® platform.

Primary outcome measure participants’ level of PTSS was measured using EGS-R [31].

Predictor variables:

(a) **Temporality:** baseline and 12-month follow-up.
(b) **Psychological and coping.**
  - Burnout: Maslach Burnout Inventory General Survey (MBI-GS). The Spanish version of the MBI-GS was used to measure the presence of burnout symptomatology through the scores of its subscales: emotional exhaustion (α= 0.84), depersonalization (α= 0.41), and personal fulfilment (α= 0.73) [32]. These subscales consist of nine, five, and eight items, respectively, measured on a seven-point Likert scale. The scale ranges from zero (never) to six (several times a week). Scores above 26 and nine for emotional exhaustion and depersonalization, respectively, and below 31 for personal fulfilment, indicate high emotional exhaustion, high depersonalization, and low personal fulfilment, corresponding to severe burnout [33].
  - Resilience: Connor-Davidson Resilience Scale-10 EUR (CD-RISC-10EUR). This scale is a self-reported assessment of the levels of resilience. It contains ten items, which are measured via a five-point Likert scale of frequencies, valued quantitatively from zero (never) to four (most of the time). The cut-off values between low, moderate, and elevated levels of resilience are 27 and 36. Moreover, the Cronbach’s alpha value of this scale is α= 0.81 [34].
  - Perceived emotional intelligence: Trait Meta-Mood Scale (TMMS-24), The TMMS is a 24-item self-report scale those measures perceived emotional intelligence (PEI). It is a trait scale of emotional meta-awareness. Specifically, it measures the skills with which we can be aware of our own emotions, as well as our ability to regulate them. This instrument is a self-completion scale that can be applied both individually and collectively. The subject who completes the questionnaire must respond by indicating his or her degree of agreement with the expression contained in each of the items on a scale ranging from 1 (I do not agree at all) to 5 (I totally agree). The TMMS assesses three dimensions of PEI: attention to emotions, clarity of emotions, and repair of emotions. Attention to emotions refers to the ability to be aware of one’s own emotions and the emotions of others; it is composed of eight items; Cronbach’s alpha was 0.90 [35]. Clarity of emotions refers to the ability to understand and label one’s own emotions, it is composed of eight items, Cronbach’s alpha was 0.90. Repair of emotions refers to the ability to regulate one’s own emotions and use them constructively It is composed of eight items; Cronbach’s alpha was 0.86 [29].
  - Work-family conflict: Questionnaire of Work-family Interaction (SWING). It is a structured assessment instrument, in a Likert-type format from 0 to 3 according to frequency. It assesses how the work and family domains are related. The scale measures a total of 22 items and is divided into 4 subscales: Negative Work-Family Interaction (work performance due to family problems) consists of 8 items, Negative Family-Work Interaction (complications in fulfilling personal obligations, caused by lack of time) consists of 4 items, Positive Work-Family Interaction (organization and commitment of the participant at home, as the origin of achieving work goals) consists of 5 items, and Positive Family-Work Interaction (capacity and organizational skills obtained at work, which make it easier to carry out domestic obligations and responsibilities) consists of 5 items [36]. The general Cronbach’s α for the total SWING Spanish version questionnaire was 0.84. The values for each dimension range from 0.85 to 0.90. The internal consistency of the theoretically relevant variables is also strong (Cronbach’s α = 0.80) [37].
  - Dysfunctionality in daily life: Reviewed Scale for the measurement of the PTSD, following the DSM-5 (EGS-R). The clinical version of the EGS-R scale has been used to measure participants’ PTSS and dysfunctionality in their daily life related to the traumatic event. The latter assesses the various difficulties that people sometimes experience following stressful events, such as the situations lived by the HCW during the state of emergency. This is a structured assessment instrument, administered hetero-applied, in a Likert-type format from 0 to 3 according to the frequency and intensity of the symptoms. This scale consists of 21 core items (range: 0-63 points) in correspondence with the DSM-5 diagnostic criteria: 5 refer to re-experiencing symptoms (range: 0-15 points), 3 to behavioural/cognitive avoidance symptoms (range: 0-9 points), 7 to cognitive disturbances and negative mood (range: 0-21 points) and 6 to symptoms of increased activation and psychophysiological reactivity (range: 0-18 points). A symptom is considered to be present when it scores at least two points on the corresponding item. The global scale ranges from 0 to 63 points. In addition to the core symptoms of PTSD, four items have been added to assess the presence of dissociative symptoms in a complementary way due to the importance given to these symptoms in the DSM-5 and six items to assess the degree of impairment or dysfunctionality related to the traumatic event. The overall instrument showed high internal consistency (α = 0.91), as well as good discriminant (g = 1.27) and convergent validity (rbp = 0.78 with diagnosis). The results of the confirmatory factor analysis support the four DSM-5 symptom clusters. A cut-off points of 20, with a diagnostic efficiency of 82.48%, is appropriate for discriminating people with PTSD [31].
  - Stress coping strategies: Stress Coping Questionnaire (SCQ). The SCQ encompasses ways of thinking and behaving towards people that are often used to cope with problems or stressful situations. It measures all the coping mechanisms described above (see 3.4) through their respective subscales, each of which contains six items. It also uses a five-point Likert scale of frequencies, ranging from zero (never) to four (most of the time). Furthermore, its Cronbach’s alpha can range from α= 0.64 to α= 0.92 [28].
  - Professional Quality of Life Scale (ProQOL-IV): This scale has been used to evaluate the participants’ levels of compassion satisfaction. This variable is measured through a total of ten items, rated on a six-point Likert frequency scale, quantitatively scored from zero (never) to five (always). The cut-off values between low, moderate, and high levels of compassion satisfaction are 22 and 42; its Cronbach’s alpha for reliability is 0.87. For burnout the cut-off values are 18 and 27 (Reliability Cronbach’s α = 0.72). Reliability Cronbach’s alpha for Compassion Fatigue is 0.80 with the cut-off points in 8 and 17 [20,38].
(c) **Socio-demographic and occupational**: Sex, Age (years), Civil status, Household, Occupation, Educational level, Experience at the start of the SA (years), Type of contract during the SA, Workstation during the SA, Contract dedication during the SA, Level of care, Contractual service, Maximum number of patients during the SA, Number of patients in the last working journey, Positive COVID-19 laboratory test during the SA, Positive COVID-19 laboratory test after the SA, Close relatives or cohabitants tested positive for COVID-19, Risk factors for COVID-19 infection.

It should be noted that the scales constitute measures for the assessment of mental disorders. They can detect the presence of considerable risk for a specific mental disorder in individuals, but the results cannot be interpreted as clinical diagnoses. However, the use of these scales allowed the results of the study to be compared with existing evidence. The remaining variables were measured by including single-item questions in the questionnaire.

### Bias

Despite the possible influence of social desirability and individual bias in the answers provided by the participants [39], these limitations have been minimised by the use of validated questionnaires and the guaranteed anonymisation of the participants.

### Study size

A minimum sample size necessary for an effect size d = 0.35 [40] of between 68 and 105 cases in total was estimated in a repeated measures ANOVA with two observations over time (two repeated measures), one factor with between 2 and 5 groups and its corresponding interaction over time, a type I error probability α = 0.05, and a power 1-β = 0.80.

### Statistical methods

All analyses were performed on all available records. We conducted no permutations due to the characteristics of the sample. A statistical significance level of p < 0.05 was established at every stage of the analysis.

Bivariate analysis was performed in between the sociodemographic and occupational variables, and PTSS scale at baseline using one-way ANOVA and two sample t-test. To analyze changes in PTSS scores between the baseline and the 12-month follow-up, linear mixed models were fitted. In the first step, the models included the effect of the variable of interest (factor), the effect of time, and the interaction between time and the factor. In the second step, the models were refitted without the interaction term when it was not statistically significant.

A generalised linear model (GLM) was performed, considering the EGS-R scale as a dependent variable, and socio-demographic (sex, age, household), occupational (occupation, contractual service, level of care, workstation during the SA, type of contract during the SA), psychological, and coping factors as explanatory. Only the significant results are shown in the final adjusted model. The effect of confounders was minimised by including all available variables that prior literature had identified as influencing the levels of PTSS. Furthermore, interaction effects and confounding factors were explored by including each variable in a stepwise manner. Once the final model was obtained, it was checked that none of the independent variables that had not been included significantly influenced the model and that there were not any interactions within the independent variables included.

### Ethics approval and consent to participate

The study was approved by the Instituto de Salud Carlos III Ethics Research Committee (CEI PI 80_2020-v3). The data were fully anonymized by the PI before investigators accessed them. Every effort has been made to ensure that the IMPRESSIONA study complies with all applicable ethical standards. Therefore, the study abides by the Declaration of Helsinki, revised in 2013 [41], and the UNESCO Declaration on the Genome and Human Rights [42]. Therefore, participation in the study is not expected to cause any harm or negative side effects to the participants, as there are no previous records of this in the literature. However, as noted above, participants with a possible post-traumatic stress disorder were excluded from the study to avoid increasing their burden.

Before recruitment began, IMPRESIONA was granted ethical clearance from all the relevant ethics committees of the participating and collaborating institutions for the collection and management of data by all members of the research team. Moreover, all the participants signed an informed consent form before their inclusion and were aware that they could withdraw from the study at any point and request the elimination of their data. The records used to develop this secondary data analysis were previously anonymized, meaning that only IMPRESIONA’s principal investigator had access to the participants’ identifiable characteristics.

## Results

### Participants

After removing the duplicate entries and those of participants with EGS-R values consistent with possible PTSD, the total number of participants was 428 (Table 1). The sample adherence to the study follow-up was 42.06%. There were no significant differences between the groups except for their age, years of experience, contractual service, and workload (S1 Table).

**Table 1:** Sociodemographic and employment baseline characteristics.

Variables	Overall n (%)
**Sex**	
*Man*	73 (17.6)
*Woman*	340 (81.9)
*Other*	2 (0.5)
	
**Civil status**	
*Single*	106 (25.0)
*Married/Domestic Partnership*	260 (61.3)
*Divorced/Separated/Widowed*	58 (13.7)
**Household**	
*With Partner/Family*	216 (50.8)
*With Dependents*	159 (37.4)
*Other*	50 (11.8)
**Occupation**	
*Nurse*	282 (65.9)
*Doctor*	57 (13.3)
*Healthcare Assistant*	83 (19.4)
*Geriatric carer or equivalent*	6 (1.4)
**Educational level**	
*Complete studies*	78 (18.3)
*University degree*	330 (77.3)
*Doctorate*	16 (3.7)
*Other*	3 (0.7)
**Experience at the start of SA (years)**	18.10 (9.85)
**Type of contract during SA**	
*Fixed*	201 (47.1)
*Interim*	139 (32.6)
*Other*	87 (20.4)
**Workstation during SA**	
*Same service*	331 (77.5)
*Different service*	76 (17.8)
*Other*	20 (4.7)
**Contract dedication during SA**)	
*Full-time contract*	377 (88.1)
*Part-time contract*	27 (6.3)
*Unpaid part-time or full-time contract*	25 (5.9)
**Level of care**	
*Primary Care*	71 (17.6)
*Hospital*	312 (77.2)
*Social and health care institution*	*21 (5.2)*
**Contractual service**	
*Emergency Service/ Intensive Care Unit*	110 (33.0)
*COVID-19 Unit*	68 (20.4)
*Other*	155 (46.5)
	
	
***Positive COVID-19 laboratory test SA***	126 (29.6)
***Positive COVID-19 laboratory test after SA***	32 (10.7)
***Close relatives or cohabitants tested positive for COVID-19***	187 (43.9)
***Risk factors for COVID-19 infection***	59 (13.8)
	*Mean (SD)*
***Age (years)***	45.06 (9.87)
***Maximum number of patients SA***	22.27 (17.87)
***Number of patients in last working journey***	14.14 (11.56)
*SA: State of Alar;*	

### Descriptive analysis

As shown in Table 1, the mean age was 45.06 years (SD = 9.87) and most of the participants were women (81.9%). The most prevalent living situation was living with dependents (37.4%). Most participants were nurses (65.9%), with a university degree (77.3%), who worked in the hospital setting (77.2%), and had full-time (88.1%) and permanent contracts (47.1%). Additionally, 77.5% of participants were in the same position as they had before. The largest number of patients attended was 22.27 (SD = 17.87) during the SA, and 14.14 (SD = 11.56) on their most recent workday. Furthermore, 70.4% and 89.3% had not been infected with COVID-19 during or after the SA, respectively. Lastly, 86.2% had no health conditions that would put them at risk of infection.

Table 2A and Table 2B present the results obtained on each scale, regarding the psychological and coping factors of all participant groups at baseline.

**Table 2:** Psychological and coping factors baseline characteristics.

A. Numeric Scales	
Scale	Overall^1^
**Revised PTSD symptom severity scale (EGS-R)**	10.99 (7.83)
	9.00 [5.00-16.00]
**Stress Coping Questionnaire (CAE)**	
*Focalization in Problem Solution*	11.92 (5.15)
	12.00 [8.00-16.00]
*Negative Focus*	6.16 (3.28)
	6.00 [4.00-8.00]
*Positive Reappraisal*	13.99 (3.83)
	14.00 [12.00-16.00]
*Open Emotional Expression*	5.53 (3.14)
	5.00 [3.00-8.00]
*Avoidance*	9.82 (3.87)
	10.00 [7.00-12.00]
*Seeking emotional support*	10.60 (5.74)
	10.00 [7.00-15.00]
*Aspects related to their Religion*	2.58 (4.50)
	0.00 [0.00-3.00]
**Questionnaire of Work-family Interaction (SWING)**	
*Negative work-family interaction*	9.47 (3.62)
	10.00 [7.00-12.00]
*Negative family-work interaction*	1.23 (1.54)
	1.00 [0.00-2.00]
*Positive work-family interaction*	7.24 (3.25)
	7.00 [5.00-10.00]
*Positive family-work interaction*	7.26 (4.07)
	7.00 [4.00-10.00]
^1^ Mean (SD); Median [Q1-Q3].	
**B. Categoric Scales**	
**Attention to feelings**^**2**^	
*Insufficient*	204 (50.9)
*Adequate*	173 (43.1)
*Excessive*	24 (6.0)
**Emotional clarity**^**3**^	
*Need for improvement*	172 (43.0)
*Adequate*	189 (47.3)
*Excellent*	39 (9.8)
**Mood repair**^**4**^	
*Need for improvement*	134 (33.5)
*Adequate*	226 (56.5)
*Excellent*	40 (10.0)
**Resilience Scale (CD-RISC 10)**	
*Low ≤ 27*	164 (39.6)
*Moderate 28–35*	176 (42.5)
*High ≥36*	74 (17.9)
**Maslach Burnout Inventory General Survey (MBI-GS)**	
**Emotional exhaustion**	
*Low ≤ 18*	133 (32.8)
*Moderate 19–26*	103 (25.4)
*High ≥27*	169 (41.7)
**Depersonalization**	
*Low ≤ 5*	107 (27.1)
*Moderate 6–9*	145 (36.7)
*High ≥10*	143 (36.2)
**Personal accomplishment**	
*Low ≤ 30*	40 (9.9)
*Moderate 31–39*	156 (38.5)
*High ≥40*	209 (51.6)
**Professional Quality of Life (ProQOL)**	
**Secondary Trauma**	
*Low ≤ 22*	91 (22.7)
*Moderate 23–41*	245 (61.1)
*High ≥42*	65 (16.2)
**Burnout**	
*Low ≤ 22*	43 (10.7)
*Moderate 23–41*	316 (78.6)
*High ≥42*	43 (10.7)
**Compassion Satisfaction**	
*Low ≤ 22*	301 (74.7)
*Moderate 23–41*	102 (25.3)
*High ≥42*	0 (0)

^1^n (%); ^2^ Cut-off scores by sex (Men/Women): Insufficient ≤21/ ≤ 24, Adequate 23–32/25–35, Excessive ≥33/ ≥ 36; ^3^ Cut-off scores (Men/Women): Need for improvement ≤25/ ≤ 23, Adequate 26–35/24–34, Excessive ≥36/ ≥ 35; ^4^ Cut-off scores (Men/Women): Need for improvement ≤23/ ≤ 23, Adequate 24–35/24–34, Excessive ≥36/ ≥ 35.

The mean PTSS score at baseline was 10.99 (SD = 7.83), indicating a low level of posttraumatic stress symptomatology. The most prevalent coping strategies were approach-focused (e.g., positive reappraisal and seeking social support) and problem-focused, both considered positive coping strategies. Negative coping mechanisms, including emotion-focused strategies (e.g., negative focus and overt emotional expression), and avoidance-focused, were less prevalent.

Regarding family-work interactions, positive interactions were more predominant at baseline. However, HCWs’ work life had a negative influence on family interactions. TMMS-24 results indicated that approximately half of the sample (50.9%) demonstrated insufficient attention to feelings, in contrast to overall adequate levels of emotional clarity and mood repair.

Additionally, 82.1% of the sample exhibited low to moderate levels of resilience. When analyzing burnout, HCWs demonstrated moderate to high levels of emotional exhaustion (70.9%), and depersonalization (72.9%), while 51.6% reported high levels of personal accomplishment. The ProQOL-IV scale indicated the pevalence of moderate burnout (78.6%). Lastly, HCWs showed predominantly moderate to high levels of secondary trauma (77.3%) and low levels of compassion satisfaction (74.7%).

### Main outcomes

PTSS EGS-R scores reduced progressively and significantly (p = 0.001) during follow-up, from 10.99 (SD = 10.99) at baseline to 7.59 (SD = 8.93) at 12 months.

**Fig 1:**
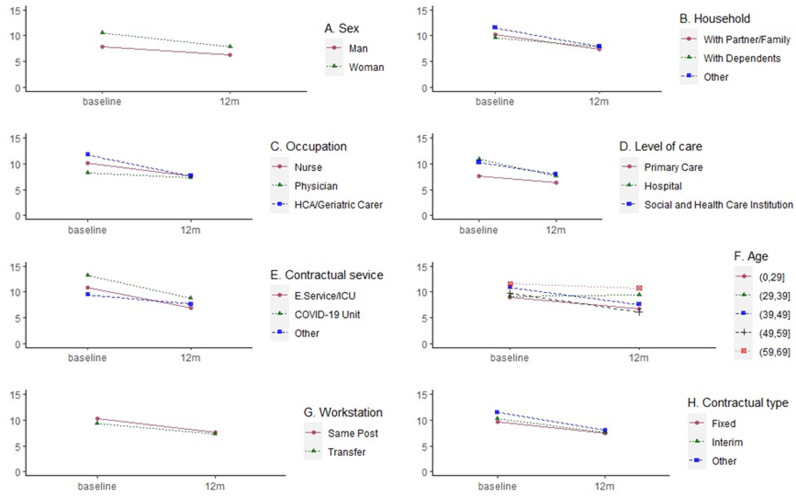
Evolution of mean values of posttraumatic stress symptomatology according to sociodemographic and occupational variables from baseline to 12-month. X-axis: Time (Baseline and 12 month). Y-axis: PTSS Score (EGS-R). HCA: Healthcare Assistant. E. Service/ICU: Emergency Service/ Intensive Care Unit.

To further identify healthcare workers (HCWs) at risk of developing post-traumatic stress symptomatology (PTSS), the influence of sociodemographic and occupational variables was analyzed both cross-sectionally at baseline (Table 3) and longitudinally over the 12-month follow-up period (Table 4, Fig 1).

**Table 3:** Baseline comparison of posttraumatic stress symptomatology according to sociodemographic and occupational variables.

Variables	Overall score^*1*^	Subcategories	N	Scores^*1*^	p value
**Sex**	10.92 (7.83)	Female	340	11.36 (7.95)	0.040^*2*^
		Male	73	8.85 (6.95)	
**Household**	10.98 (7.82)	With partner/family	216	10.88 (7.51)	0.956^*3*^
		With dependents	159	11.03 (8.04)	
		Other	50	11.22 (8.55)	
		Nurse	282	11.06 (7.50)	
**Occupation**	10.99 (7.83)	Physician	57	9.07 (8.32)	0.087^*3*^
		HCA/Geriatric carer	89	11.98 (8.36)	
**facilityLevel of care**	10.91 (7.85)	Primary Care	71	8.90 (7.23)	0.039^*3*^
		Hospital	312	11.44 (8.01)	
		Social/health care institution	21	9.81 (6.52)	
**Contractual service**	11.34 (7.92)	Emergency Service/ICU	110	12.82 (8.80)	0.004^*3*^
		COVID-19 unit	68	12.43 (7.74)	
		Other	155	9.81 (7.07)	
**Age**	10.99 (7.83)	(0,29]	27	11.33 (7.52)	0.373^*3*^
		(29,39]	94	11.16 (7.52)	
		(39,49]	162	11.64 (8.40)	
		(49,59]	114	9.73 (7.28)	
		(59,69]	31	11.42 (7.80)	
**Workstation**	11.00 (7.85)	Same service	331	11.27 (8.00)	0.165^*2*^
		Different service	76	9.88 (7.12)	
**Contractual type**	10.96 (7.82)	Fixed	201	10.43 (7.36)	0.401^*2*^
		Interim	139	11.53 (8.05)	
		Other	87	11.31 (8.46)	

HCA: Healthcare assistant; ICU: Intensive Care Unit; ^1^Mean (SD); ^2^Two Sample t-test; ^3^One-way ANOVA.

**Table 4:** Linear mixed models. evolution of posttraumatric stress symptomatology scores according to socio-demographic and occupational variables, from baseline to 12 months.

Model	Coef	95% CI	p
Sex			0.112
Female			
Male	-2.12	-4.72, 0.50	0.112
Time	-2.41	-3.98, -0.83	0.003
Age			0.553
0-29			
30-39	1.39	-3.50, 6.27	0.575
40-49	1.34	-3.13, 5.81	0.555
50-59	0.02	-4.51, 4.54	0.995
60-69	3.27	-2.35, 8.89	0.253
time	-2.57	-4.11, -1.04	0.001
Household			0.857
With partner/family			
With dependents	-0.08	-2.28, 2.11	0.941
Other	0.88	-2.54, 4.30	0.612
Time	-2.57	-4.11, -1.04	0.001
Occupation			0.555
Nurse			
Physician	-1.08	-3.95, 1.78	0.456
HCA/Geriatric carer	0.83	-1.84, 3.51	0.539
Time	-2.57	-4.11, -1.04	0.001
Contractual type			0.702
Fixed			
Interim	0.37	-1.92, 2.67	0.747
Other	1.22	-1.66, 4.10	0.402
Time	-2.57	-4.10, -1.04	0.001
Workstation			0.649
Same service			
Different service	0.63	-2.10, 3.37	0.649
Time	-2.55	-4.15, -0.95	0.002
Level of care			0.240
Primary care			
Hospital	2.28	-0.39, 4.94	0.093
Social/health care institution	2.17	-2.62, 2.16	0.373
Time	-2.72	-4.32, -1.13	0.001
Contractual.service			0.246
Emergency service/ICU			
COVID-19 unit	2.13	-1.17, 5.44	0.204
Other	-0.31	-3.17, 2.56	0.832
Time	-3.09	-4.91, -1.26	0.001

HCA: healthcare assistant; ICU: intensive care unit.

At baseline, significant differences were found between men and women (p = 0.040), with women reporting higher PTSS scores. However, these differences were not significant during the follow-up period (p = 0.112) (Fig 1A). Regarding household composition, no significant differences in PTSS were found either at baseline (p = 0.956) or at follow-up (p = 0.857) (Fig 1B).

No statistically significant differences were observed between professional categories at any time point. However, healthcare assistants (HCA), geriatric carers, and nurses showed higher PTSS scores than physicians at baseline, though these differences did not persist over time (Fig 1C).

Regarding the level of care, significant differences were observed at baseline (p = 0.039), with hospital and social healthcare workers reporting higher PTSS scores compared to those in primary care. These differences were no longer present during the follow-up period (p = 0.240) (Fig 1D). Within the hospital setting, HCWs working in Emergency Services/ICUs and COVID-19-specific units reported significantly higher PTSS scores at baseline (p = 0.004), but these differences were not observed at follow-up (p = 0.246) (Fig 1E).

These findings are consistent with the results of the linear mixed models (Table 4), in which time was the only variable consistently associated with PTSS scores. A significant decrease in PTSS was observed over the 12-month period (e.g., Coef. = ‐2.57; 95% CI: ‐4.11 to ‐1.04; p = 0.001), regardless of sex, age, household type, professional role, contract type, workstation, level of care, or service type. No significant interaction effects between time and any variable were found, and thus were removed from the final models which were refitted without these effects.

In the latter GLM model, work-family negative influence (0.477, p < 0.001), avoidant coping (0.386, p < 0.001), mood repair (‐0.164, p = 0.021), emotional exhaustion (0.172, p < 0.001), depersonalization (‐0.351, p < 0.001), attention to feelings (0.186, p = 0.004), emotional clarity (‐0.177, p = 0.021), and having changed service (‐2.04, p = 0.017), explained up to 25.5% of the variations observed in PTSS (Table 5).

**Table 5:** Estimation of the coefficients of a generalised linear model of posttraumatic stress symptomatology.

Variables	β	Std. error	CI 95%		p value
(Intercept)	7.44	2.33	2.87	12.01	0.002
Work-family negative influence	0.477	0.1	0.28	0.67	<0.001
Avoidant coping	0.386	0.096	0.20	0.57	<0.001
TMMS: Mood Repair	-0.164	0.071	-0.30	-0.02	0.021
MBI: Emotional Exhaustion	0.172	0.047	0.08	0.26	<0.001
MBI: Depersonalization	-0.351	0.092	-0.53	-0.17	<0.001
Different service	-2.04	0.849	-3.70	-0.38	0.017
TMMS: Attention to Feelings	0.186	0.063	0.06	0.31	0.004
TMMS: Emotional Clarity	-0.177	0.076	-0.33	-0.03	0.021

Std. Error: Standard Error; TMMS: Trait Meta Mood Scale; MBI: Maslach Burnout Inventory, Type contract (other): working in a different post during the state of alarm; CI 95%: 95% confidence.

## Discussion

### Key results and interpretation

The predominant HCW profile in this study was a middle-aged female nurse, working full time with a fixed contract in a hospital, who had not been infected with COVID-19 and was not part of the high-risk population.

Despite presenting generally low to moderate resilience levels, the participants of the present study employed healthy coping mechanisms during the outbreak, with the highest mean scores observed for positive reappraisal (mean = 13.99), seeking emotional support (mean = 10.60), and focusing on problem-solving (mean = 12.15). This tendency towards adaptive coping has also been observed in studies of healthcare workers during COVID-19 [43,44], where ‘contributing to improve a situation’ was identified as the main strategy in Italy [43], and mean scores of 15.57 for problem-solving, 11.89 for positive reappraisal, and 8.16 for seeking social support were reported in Mexico [44]. These findings suggest that HCWs had prior knowledge and training on how to cope effectively with stress, which may have contributed to mitigating the psychological impact of the pandemic. In our sample, although 82.1% of participants exhibited low to moderate resilience, the strategies with the highest mean scores were positive reappraisal (mean = 13.99), problem-solving (mean = 12.15), and seeking social support (mean = 10.60), reflecting a tendency towards adaptive coping.

However, avoidance-focused strategies also showed a relatively high mean score (mean = 9.82), which was not significantly lower than those of the adaptive strategies, indicating the coexistence of adaptive and maladaptive coping. This may relate to early psychological reactions observed in HCWs. For instance, Sánchez-Sánchez et al. [5] reported that 68.3% of Spanish nurses and nursing assistants experienced anxiety and 49.6% reported depressive symptoms during the first wave of the pandemic, though both rates decreased significantly in the second wave (p < 0.001). As highlighted by Jiménez-Giménez et al. [10], many professionals tended to postpone the processing of emotional distress during the initial phases of the crisis, prioritizing care delivery. This tendency may explain our participants’ limited attention to emotional states, with 50.9% showing insufficient emotional awareness despite adequate clarity and mood repair. At baseline, specific psychological factors that significantly influenced PTSS levels included high emotional exhaustion, increased use of avoidant coping strategies, and low levels of emotional clarity. These patterns suggest that difficulties in emotion regulation and reliance on maladaptive coping mechanisms played a central role in early PTSS expression.

Positive interactions between family and work life predominated, meaning that organisational skills and commitment at home and at work reinforced the abilities of professionals to perform their duties and responsibilities more easily [36]. However, high scores in the negative work-family interaction indicated decreased job performance due to family problems.

Similar to personal accomplishment, compassion satisfaction has been described as a protective factor against burnout symptomatology during the first COVID-19 outbreak in Spain. A study of social workers in Israel found a negative correlation between compassion satisfaction, burnout, and secondary trauma [45]. In our sample, most participants reported low levels of compassion satisfaction, while a smaller portion presented moderate levels, and no one reached high scores. These patterns were consistent with elevated levels of emotional exhaustion and depersonalization. These findings pose a major threat to health standards, as burnout is associated with lower quality of care [11] higher intention to leave (adjusted OR = 1.29) and emotional exhaustion (adjusted OR = 1.26) [46], and has been linked to anxiety (23.2%) and depression (22.8%) among HCWs during the pandemic [18].

As in previous research after the first wave of COVID-19 [8], PTSS decreased during follow-up on socio-economic and occupational characteristics. The higher COVID-19 severity at the beginning of the pandemic and the fear of contagion could explain the baseline results [23]. However, PTSS decline could also be explained by the initial high prevalence of acute stress, which shares similarities and can be confounded with PTSS. In a large study conducted during the COVID-19 peak in New York, 57% of healthcare workers screened positive for acute stress symptoms [47].

The fact that living with family, a partner, or dependents was described as a risk factor for PTSS at the beginning of follow-up is supported by previous literature. In our sample, more than one third of participants reported living with dependents, and negative family-work interactions were significantly associated with higher PTSS levels at baseline (adjusted β = 0.477, p < 0.001), contributing to 25.5% of the explained variance. In a Spanish national study, nearly half of HCWs screened positive for at least one mental disorder, with acute stress being among the most prevalent conditions, and fear of infecting family members cited as a major stressor [48]. This behaviour was likely driven by concerns about the health and economic consequences of contagion for their families, workplaces, and society [3]. However, the absence of significant association during the follow-up period, might be explained by of the protective role of perceived social support, which has shown inverse associations with depressive symptoms, psychological distress, and suicidal ideation in HCWs [48]. Nonetheless, prolonged isolation may have weakened this protective effect over time, as HCWs increasingly reported reduced access to informal emotional support networks [18].

Although only at baseline, HCAs showed higher PTSS levels than other HCWs. Like nurses, they provide more direct and prolonged care to patients, increasing their vulnerability to trauma-related symptoms. This pattern is consistent with our findings, which showed higher PTSS scores among HCAs and nurses than among physicians or other staff categories at the start of follow-up. A meta-analysis supports these results, reporting the highest post-pandemic PTSD prevalence among HCWs (27%), compared to infected patients (24%) and the general population (19%) [22]. Nurses and women have also been consistently identified as particularly vulnerable subgroups [26]. Despite the decrease in mortality and hospitalization rates compared to the first outbreak, HCWs working at hospitals were still at significant risk of infection and in closer contact with patients of higher severity than other levels of care [1]. This may explain why HCWs in hospital settings—particularly those working in ERs, ICUs, and COVID-19-specific units—were more vulnerable to higher PTSS levels at baseline. While our findings showed a trend in this direction, no statistically significant differences were observed during the follow-up period.

The GLM model showed that the variations in the baseline levels of PTSS were explained by up to 25.5% through the influence of avoidant coping, emotional intelligence levels, having changed workstation, work-family negative influence, and emotional exhaustion and depersonalization. This underscores the importance that psychological and coping factors have on the PTSS levels, in comparison with other occupational and sociodemographic determinants. In line with our findings, Sobregrau et al. [49] also identified psychological burden as being significantly influenced by emotional and behavioural risk factors, such as stress-related medication, overworking, and substance use, rather than by structural workplace characteristics. While our results offer an initial insight into the psychological functioning and needs of HCWs, further research is required to better understand the interplay of these and other variables in shaping mental health outcomes and to inform the development of tailored, evidence-based interventions.

Associations were only found between non-adaptive strategies, such as avoidant coping, and more severe symptomatology, reinforcing the fact that negative coping mechanisms enhance the development of negative mental health outcomes [23]. Moreover, while emotional management showed to be a protective factor, negative work-family interaction and emotional exhaustion, were risk factors. These associations, combined with the fact that the identified risk factors were prevalent in the sample, reinforce prior evidence suggesting that interventions targeting PTSS should focus on improving positive coping, emotion regulation, social support, and the capacity of meaning-making and giving life a purpose [50].

Whilst high depersonalization is one of the keys defining terms for the presence of burnout, which has been associated with an increased risk of PTSS in healthcare professionals [15], the model presented it as a protective factor. This apparent contradiction had already been observed in a study conducted in Spain during the same period, which found that professionals with higher levels of depersonalization reported lower levels of emotional exhaustion [8]. This suggests that, in high-demand contexts and in the face of prolonged exposure to suffering, depersonalization may act as a temporary defence mechanism, facilitating emotional distancing and buffering the immediate psychological impact. In this sense, our findings would be consistent with the hypothesis that, under certain conditions, depersonalization plays an adaptive role in the short term, despite its traditionally negative connotation.

Moreover, despite the key role that having adequate levels of emotional intelligence plays in mental health [51], the presence of excessive levels of attentiveness to feelings has been described as a risk factor in certain stress and anxiety contexts, especially when not accompanied by emotional clarity and effective mood repair strategies. Our findings align with this view, as attentiveness to feelings appeared as a risk factor for PTSS when not balanced by the ability to regulate or interpret those emotional experiences. This pattern is consistent with previous evidence indicating that individuals with high attention to feelings but low clarity and repair are more vulnerable to distress and clinical symptoms [52]. This could explain its role as a risk factor in the described model.

The fact that changing service from their usual practice during the SA was a protective factor could be justified because the two risk factors for long-term mental health problems following exposure to trauma are lack of social support and exposure to stressors during the recovery period [53].

These results suggest that while certain demographic and occupational factors were associated with higher PTSS scores at the onset of the pandemic, the psychological impact appeared to converge across groups over time, highlighting the general trend of PTSS improvement during the 12-month follow-up.

Although further research is needed to confirm these associations, this study suggests that the development of holistic interventions and policies that focus on psychological and coping factors, is essential to meet the current and future health needs of the Spanish HCWs. The importance of addressing them goes beyond improving their well-being, as it also lies in the negative consequences that not doing so could have on the health levels of the general population.

### Implications for practice and policy

These findings have several practical implications for healthcare systems and mental health policy. First, the identification of avoidant coping, emotional exhaustion, and negative work-family interaction as key risk factors for PTSS highlights the need to implement institutional strategies focused on improving emotional regulation skills and promoting adaptive coping styles among HCWs. Second, the protective role of factors such as positive coping, emotional clarity, and social support underlines the importance of strengthening peer support networks, supervision, and programs to improve emotional intelligence in clinical settings. Third, the observation that certain occupational roles (e.g., HCAs and nurses) were more vulnerable to psychological distress in the early stages suggests that mental health resources should be prioritized for professionals exposed to high emotional demand. Finally, regular psychological monitoring and the incorporation of tailored, low-burden mental health interventions into routine clinical practice could contribute to preventing long-term deterioration and improving workforce sustainability during future health crises.

### Strengths, limitations, and future research

The obtained results, their discussion, and the reached conclusions should be interpreted with the following considerations in mind. Firstly, it should be noted that the sample attrition analysis showed no significant differences in the characteristics of the participants throughout the study, despite the substantial decrease in the sample size during follow-up. Therefore, the variations observed in the main study variables cannot be directly attributed to changes in sample size. Nonetheless, to maximize the utility of the available data, all analyses have been performed with all available and eligible records.

Furthermore, the external validity of the results may present some shortcomings due to the sampling method and inclusion criteria of IMPRESIONA. The data used for this dissertation are the result of a non-random sampling method, which increases the risk of participation bias [54]. However, the characteristics of the sample regarding variables such as gender and profession do not show major differences from those of the Spanish HCWs [55]. Nonetheless, due to the inclusion criteria of IMPRESIONA, HCWs who were identified as a population at risk for SARS-CoV-2 infection, and who were thereby removed from the clinical practice with patients infected or suspected to be infected by SARS-CoV-2 were not included in the sample. Moreover, those who presented possible PTSD were also excluded from the study for ethical reasons. This adds to the possible influence on sample attrition of the ‘healthy survivor effect’, where less psychologically stable populations are more likely to withdraw during the follow-up period [56]. Therefore, the results have been obtained from a sample of healthy and more psychologically stable HCWs. An example of how this might have influenced the results is the reported positive association between the absence of pre-existing mental health problems and better levels of mental health in HCWs during the pandemic [17].

Despite the possible influence of social desirability and individual bias in the answers provided by the participants [39,57], these limitations have been minimised by both the use of validated questionnaires and the guaranteed anonymisation of the participants.

Additionally, it is important to note that the scales are screening measures of mental disorders. While they are valid for identifying individuals with a substantial risk of a particular 34 mental disorder, the results cannot be interpreted as clinical diagnoses. However, the use of these specific measures allows for the comparison of the study results with existing evidence.

The use of multivariate regression models made it possible to study the simultaneous effect of all independent variables on the dependent variables, better reflecting how these factors interact in the real-life context. Additionally, as the temporality of the subscales of the main independent variables always preceded that of the dependent variables, the models allowed minimising the effect of reverse causality. However, as different subscales with differing cut-off points were used, the models did not allow comparison of the individual influence of the independent variables.

Notwithstanding the use of validated questionnaires, this quantitative research has limitations in understanding the nuances and intricacies of HCW’s experiences, needs, and perspectives about which support they would find most useful and when. Hence, due to the negative impact that failure to tailor interventions to the needs of HCW can have on their well-being and that of the population [8,58], further research and larger involvement of the HCW in decision-making and management is required [26]. This would enable timely and more appropriate support to be provided to the HCW [10]. While the use of qualitative research would also be beneficial [26], as it requires more time from the participants, it may not be appropriate to use this methodology until there is a significant decrease in the burden of the pandemic [56].

Despite the limitations described above, this study has contributed to the early detection of emerging mental health problems. This is especially important as studies show that the two risk factors for long-term mental health problems after exposure to a trauma are, the lack of social support, and exposure to stressors during the period of recovery [53]. Hence, this reinforces the importance of actively monitoring the health of HCWs, supporting them, and providing them with specific treatment to improve their mental health and ensure recovery [22].

Furthermore, due to the paucity of longitudinal studies focusing on the impact of the COVID-19 pandemic on the mental health of HCWs [16], this study represents a pivotal step in the generating of hypotheses that contribute to a better understanding of the phenomenon. This research contributes to the limited body of evidence aimed to understand how PTSS and burnout have evolved throughout the COVID-19 pandemic and provides critical insights into the psychological adjustment among HCWs. Furthermore, it offers evidence that may support the development of preventive, targeted, and management strategies for current and future public health crises, thereby helping to maintain the efficiency of health systems. It is also hoped that the findings of this study will contribute to raising awareness about the consequences of using negative coping mechanisms and promoting healthier responses, thereby improving the health, efficiency, and productivity of the HCWs [59].

## Conclusions

The COVID-19 pandemic has triggered an unprecedented health crisis that has caused health workers to work under extremely difficult conditions, which have had a negative impact on their mental health. Nonetheless, the levels of posttraumatic stress symptomatology have decreased significantly following the state of alarm.

Socio-demographic and occupational factors seemed to influence the initial levels of posttraumatic stress symptomatology, but when studied in conjunction with psychological and coping characteristics, their influence became non-significant. Although coping and psychological factors, including burnout levels, work-family influence, and emotional management, have been shown to play a key role in modulating PTSS levels, the results highlight the additional influence of unexplored factors.

## Supporting information

Table S1 Comparison of the sociodemographic and occupational sample baseline characteristics of the participants from the 3 follow up groups (No follow-up, 6-months, 12-month). (DOCX)
