# Supplementary material for: Factors modulating the impact of the COVID-19 pandemic on posttraumatic stress symptomatology of the Spanish healthcare workers: A cohort study
Source: PLoS One. 2025 Jun 16;20(6):e0323777. doi: 10.1371/journal.pone.0323777 (PMC12169526; doi:10.1371/journal.pone.0323777)
Supplement: Table S1 — (DOCX) [file pone.0323777.s001.docx]

**Table S1.** Comparison of the sociodemographic and occupational sample baseline characteristics of the participants from the 3 follow up groups (No follow-up, 6-months, 12-month)

| **Variables^1^** | **No follow-up**  N = 122 | **6-months**  N = 126 | **12-months**  N = 180 | **Overall**  N = 428 | **p value^2^** |
| --- | --- | --- | --- | --- | --- |
| **Sex** |  |  |  |  | 0.374 |
| *Man* | 17 (14.3%) | 21 (16.9%) | 35 (20.6%) | 73 (17.7%) |  |
| *Woman* | 102 (85.7%) | 103 (83.1%) | 135 (79.4%) | 340 (82.3%) |  |
| **Age (years)** | 45.06 (9.87) | 43.34 (10.32) | 44.79 (10.02) | 46.42 (9.29) | 0.026 |
| **Civil status** |  |  |  |  | 0.577 |
| *Single* | 37 (30.6%) | 29 (23.2%) | 40 (22.5%) | 106 (25.0%) |  |
| *Married/Domestic partnership* | 68 (56.2%) | 79 (63.2%) | 113 (63.5%) | 260 (61.3%) |  |
| *Divorced/separated/widowed* | 16 (13.2%) | 17 (13.6%) | 25 (14.0%) | 58 (13.7%) |  |
| **Household** |  |  |  |  | 0.684 |
| *With partner/family* | 57 (47.1%) | 68 (54.8%) | 91 (50.6%) | 216 (50.8%) |  |
| *With dependents* | 47 (38.8%) | 42 (33.9%) | 70 (38.9%) | 159 (37.4%) |  |
| *Other* | 17 (14.0%) | 14 (11.3%) | 19 (10.6%) | 50 (11.8%) |  |
| **Occupation** |  |  |  |  | 0.679 |
| *Nurse* | 76 (62.3%) | 87 (69.0%) | 119 (66.1%) | 282 (65.9%) |  |
| *Doctor* | 19 (15.6%) | 12 (9.5%) | 26 (14.4%) | 57 (13.3%) |  |
| *Healthcare assistant* | 26 (21.3%) | 24 (19.0%) | 33 (18.3%) | 83 (19.4%) |  |
| *Geriatric carer or equivalent* | 1 (0.8%) | 3 (2.4%) | 2 (1.1%) | 6 (1.4%) |  |
| **Educational level** |  |  |  |  | 0.684 |
| *Complete studies* | 24 (19.8%) | 24 (19.0%) | 30 (16.7%) | 78 (18.3%) |  |
| *University degree* | 93 (76.9%) | 96 (76.2%) | 141 (78.3%) | 330 (77.3%) |  |
| *Doctorate* | 4 (3.3%) | 5 (4.0%) | 7 (3.9%) | 16 (3.7%) |  |
| *Other* | 0 (0%) | 1 (0.8%) | 2 (1.1%) | 3 (0.7%) |  |
| **Experience at the start of SA (years)** | 18.10 (9.85) | 16.92 (10.01) | 16.80 (10.12) | 19.81 (9.34) | 0.009 |
| **Contract dedication during SA** |  |  |  |  | 0.464 |
| *Full-time contract* | 105 (86.8%) | 113 (91.1%) | 155 (86.6%) | 373 (88.0%) |  |
| *Part-time contract* | 10 (8.3%) | 6 (4.8%) | 10 (5.6%) | 26 (6.1%) |  |
| *Unpaid part-time or full-time contract* | 6 (5.0%) | 5 (4.0%) | 14 (7.8%) | 25 (5.9%) |  |
| **Type of contract during SA** |  |  |  |  | 0.340 |
| *Fixed* | 52 (42.6%) | 58 (46.4%) | 91 (50.6%) | 201 (47.1%) |  |
| *Interim* | 40 (32.8%) | 38 (30.4%) | 61 (33.9%) | 139 (32.6%) |  |
| *Other* | 30 (24.6%) | 29 (23.2%) | 28 (15.6%) | 87 (20.4%) |  |
| **Workstation during SA** |  |  |  |  | 0.932 |
| *Same post* | 92 (76.0%) | 97 (77.0%) | 142 (78.9%) | 331 (77.5%) |  |
| *Transfer* | 22 (18.2%) | 24 (19.0%) | 30 (16.7%) | 76 (17.8%) |  |
| *Other* | 7 (5.8%) | 5 (4.0%) | 8 (4.4%) | 20 (4.7%) |  |
| **Level of care** |  |  |  |  | 0.693 |
| *Primary Care* | 71 (17.6%) | 16 (13.7%) | 22 (19.0%) | 33 (19.3%) |  |
| *Hospital* | 312 (77.2%) | 96 (82.1%) | 88 (75.9%) | 128 (74.9%) |  |
| *Social and health care institution* | 21 (5.2%) | 5 (4.3%) | 6 (5.2%) | 10 (5.8%) |  |
| **Contractual service** |  |  |  |  | 0.008 |
| *Same post* | 92 (76.0%) | 97 (77.0%) | 142 (78.9%) | 331 (77.5%) |  |
| *Transfer* | 22 (18.2%) | 24 (19.0%) | 30 (16.7%) | 76 (17.8%) |  |
| *Other* | 7 (5.8%) | 5 (4.0%) | 8 (4.4%) | 20 (4.7%) |  |
| **Maximum number of patients SA** | 22.27 (17.87) | 18.74 (15.40) | 24.08 (16.35) | 23.37 (20.07) | 0.035 |
| **Number of patients in last working journey** | 14.14 (11.56) | 11.66 (10.73) | 15.72 (11.31) | 14.78 (12.06) | 0.017 |
| **Positive COVID-19 test during SA** | 126 (29.6%) | 33 (27.5%) | 37 (29.4%) | 56 (31.1%) | 0.811 |
| **Positive COVID-19 test after SA** | 32 (10.7%) | 9 (10.3%) | 9 (10.1%) | 14 (11.3%) | 0.979 |
| **Close relatives or cohabitants tested positive for COVID-19** | 187 (43.9%) | 53 (44.2%) | 51 (40.5%) | 83 (46.1%) | 0.622 |
| **Risk factors for COVID-19 infection** | 59 (13.8%) | 18 (14.8%) | 21 (16.7%) | 20 (11.1%) | 0.622 |
| *SA: State of Alarm.*  *No follow-up: characteristics of no follow-up group.*  *6-months: baseline characteristics of 6-month follow-up group.*  *12-months: baseline characteristics of 12-month follow-up group.*  *^1^ n (%); Mean (SD).*  *^2^ Chi Squared/Fisher’s Exact Test; One-way ANOVA.* | | | | | |
